# Effects of acetazolamide on control of breathing in sleep apnea patients: Mechanistic insights using meta‐analyses and physiological model simulations

**DOI:** 10.14814/phy2.15071

**Published:** 2021-10-26

**Authors:** Christopher N. Schmickl, Shane Landry, Jeremy E. Orr, Brandon Nokes, Bradley A. Edwards, Atul Malhotra, Robert L. Owens

**Affiliations:** ^1^ Division of Pulmonary, Critical Care and Sleep Medicine University of California, San Diego (UCSD) La Jolla California USA; ^2^ Department of Physiology Sleep and Circadian Medicine Laboratory School of Biomedical Sciences and Biomedical Discovery Institute Monash University Melbourne Victoria Australia; ^3^ Turner Institute for Brain and Mental Health Monash University Melbourne Victoria Australia

**Keywords:** acetazolamide, respiration, sleep apnea syndromes

## Abstract

Obstructive and central sleep apnea affects ~1 billion people globally and may lead to serious cardiovascular and neurocognitive consequences, but treatment options are limited. High loop gain (ventilatory instability) is a major pathophysiological mechanism underlying both types of sleep apnea and can be lowered pharmacologically with acetazolamide, thereby improving sleep apnea severity. However, individual responses vary and are strongly correlated with the loop gain reduction achieved by acetazolamide. To aid with patient selection for long‐term trials and clinical care, our goal was to understand better the factors that determine the change in loop gain following acetazolamide in human subjects with sleep apnea. Thus, we (i) performed several meta‐analyses to clarify how acetazolamide affects ventilatory control and loop gain (including its primary components controller/plant gain), and based on these results, we (ii) performed physiological model simulations to assess how different baseline conditions affect the change in loop gain. Our results suggest that (i) acetazolamide primarily causes a left shift of the chemosensitivity line thus lowering plant gain without substantially affecting controller gain; and (ii) higher controller gain, higher paCO_2_ at eupneic ventilation, and lower CO_2_ production at baseline result in a more pronounced loop gain reduction with acetazolamide. In summary, the combination of mechanistic meta‐analyses with model simulations provides a unified framework of acetazolamide’s effects on ventilatory control and revealed physiological predictors of response, which are consistent with empirical observations of acetazolamide's effects in different sleep apnea subgroups. Prospective studies are needed to validate these predictors and assess their value for patient selection.

## Graphical abstract

(a) Based on mechanistic meta‐analyses in patients with sleep apnea, acetazolamide primarily causes a left‐shift of the chemosensitivity line thus lowering plant gain without substantially affecting controller gain; the net effect is a relative reduction in overall loop gain similar to the reduction in plant gain. (b) Based on model simulations, a higher controller gain, higher paCO_2_ at eupneic ventilation, and lower CO_2_ production at baseline each result in a more pronounced loop gain reduction with acetazolamide, and thus may predict response to acetazolamide.

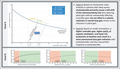

## 1 INTRODUCTION

Acetazolamide is a carbonic anhydrase inhibitor that causes bicarbonaturia, thereby producing metabolic acidosis and a concomitant increase in ventilation (Swenson, 1998). Acetazolamide reduces ventilatory instability or “high loop gain” (for more details, see the “Results of Meta‐Analyses” section below Edwards et al., 2012, which is the pathophysiological mechanism underlying most types of central sleep apnea including high altitude periodic breathing and Cheyne Stokes respiration (CSA‐CSR); (Sands et al., 2017) but loop gain is also a key contributor to obstructive sleep apnea (OSA) pathogenesis (Eckert et al., 2013; Orr et al., 2017). Based on comprehensive meta‐analyses, we recently found that acetazolamide can substantially improve both OSA and CSA (Schmickl et al., 2020): Overall, based on *study*‐*level* data, the apnea‐hypopnea index (AHI) improved by 38% (95% CI: 31–45). AHI reductions were greater in studies administering higher doses of acetazolamide (at least up to ~500 mg/day) and tended to be more pronounced in studies focusing on CSA (especially high altitude and heart failure‐related CSA). However, based on *patient*‐*level* data, interindividual changes in AHI varied widely and were not well explained by dose or sleep apnea type. This heterogeneity in the efficacy of acetazolamide underscores the need to identify predictors of response in individuals.

Based on pathophysiological considerations, one would expect that for a given sleep apnea patient, the therapeutic response (change in AHI) is driven by:

1. How much does acetazolamide alter loop gain?
2. How high is loop gain at baseline?
3. Are there any other causes of sleep apnea (unaltered by acetazolamide)?

In fact, one study demonstrated a strong positive correlation between the change in loop gain and the change in AHI (*r* = 0.63, *p* = 0.001; Terrill et al., 2015) To aid with patient selection for long‐term trials and clinical care, our objective for the current study was to understand better the factors that determine the change in loop gain following acetazolamide in human subjects with sleep apnea. Our recently published systematic review (Schmickl et al., 2020) focused on acetazolamide’s effect on clinical outcomes (e.g., apnea‐hypopnea index, blood pressure, etc.) in patients with sleep apnea, but in the process, we also collected data on acetazolamide's effect on control of breathing parameters (e.g., controller/plant gain, CO_2_ production, etc.—for details see below). For the current study, we used these unpublished data to (i) perform meta‐analyses clarifying the mechanisms through which acetazolamide lowers loop gain and, based on these results, (ii) to perform physiological model simulations to assess how different baseline conditions affect the relative change in loop gain (i.e., assess predictors of response).

## 2 METHODS

### 2.1 Conceptual framework

Loop gain is an engineering term that describes a negative feedback control system (Dempsey, 2019; Orr et al., 2017; Schmickl & Malhotra, 2020; Terrill et al., 2015; Younes, 2008). In the setting of ventilatory control, loop gain describes the interactions between the chemoreceptors (controller) and the lungs (plant) aiming to keep paCO_2_ stable around ~40 mmHg.

loop gain∝controller gain×plant Gain.

Thus, if the controller and/or plant gain are elevated, then loop gain is high, which means that any minor perturbation in breathing (e.g., hypopnea due to upper airway collapse or hyperpnea following arousal from sleep) can lead to markedly fluctuating levels of paCO_2_, ventilation and upper airway dilator tone resulting in unstable breathing (Badr et al., 1995). In CSA, this instability can lead to periodic breathing and in OSA to repetitive obstructive events (“apnea begets apnea”). Conversely, lowering either loop gain component is expected to stabilize breathing and thus improve OSA/CSA as has been demonstrated in multiple studies (Schmickl et al., 2020).

**FIGURE 1:**
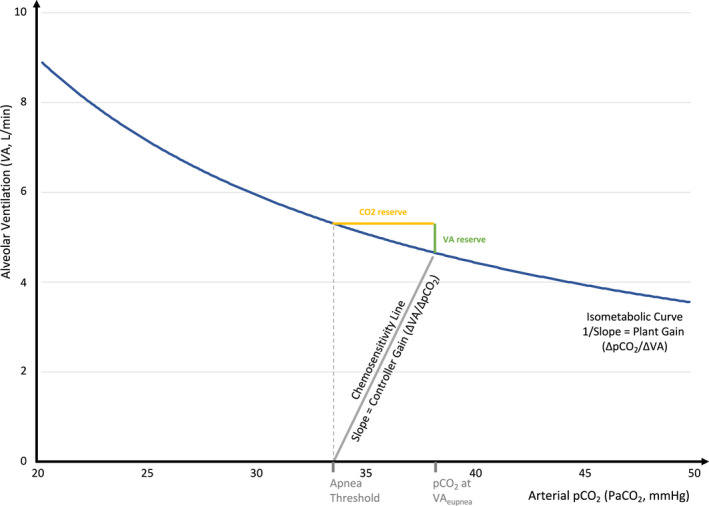
Control of breathing model. Key parameters were defined based on data from the control groups shown in Table 1 (i.e., assuming CO_2_ production = 206 ml/min, paCO_2_ at VA_eupnea_ = 38.2 mmHg, and paCO_2_ at the apnea threshold = 33.5 mmHg)

The steady‐state control of the breathing model is shown in Figure 1 that illustrates how these two loop gain components interact: (Dempsey, 2019).

#### 2.1.1 Controller gain

Chemosensitivity or “controller gain” describes the change in ventilation in response to changes in blood gases based on information from both central and peripheral chemoreceptors. In most (normoxic) conditions, the arterial paCO_2_ is the primary stimulus for ventilation (Eckert & Butler, 2017). The ventilatory response to changes in CO_2_ (VRCO_2_) can be determined experimentally by measuring ventilation in response to varying paCO_2_ levels in spontaneously breathing subjects (ΔVA/ΔpaCO_2_). Similarly, one can determine the ventilatory response to changes in arterial pO_2_ (VRO_2_; ΔVA/ΔpaO_2_), but in general, oxygen increases ventilation only if hypoxia is severe (i.e., paO_2_ < 50–60 mmHg), for example, at high altitude (Douglas et al., 1982). In addition, hypoxia increases the VRCO_2_ (O_2_–CO_2_ interaction); (Lloyd et al., 1958; West & KLuks, 2016). Thus, in normoxic conditions, the controller gain equals the VRCO_2_ (i.e., ΔVA/ΔpaCO_2_), but in hypoxic conditions, such as high altitude controller gain is higher because it is a function of both the VRCO_2_ and the VRO_2_, plus hypoxia increases the VRCO_2_ itself. Unless stated otherwise, in the following, we will assume normoxic conditions. Further, we assumed that the ventilatory response to varying levels of paCO_2_ (i.e., VRCO_2_) is the same above and below eupnea (Xie et al., 2013).

#### 2.1.2 Plant gain

The isometabolic curve reflects the relationship between alveolar ventilation (VA) and alveolar carbon dioxide (pACO_2_) and is defined as:

$$\text{VA}=\left({\text{CO}}_{2}\;\text{production}\times 0.863\right)/{\text{pACO}}_{2}$$

Note that the ventilation for a given pACO_2_ depends on the metabolic CO_2_ production, hence the name *isometabolic* curve. When plotted with ventilation as the dependent variable (i.e., on the *y*‐axis as in Figure 1) then the reciprocal of the slope equals ΔpACO_2_/ΔVA. This ratio reflects how efficiently the lungs excrete CO_2_ and is known as “plant gain.” Given the hyperbolic shape of the isometabolic curve, plant gain varies depending on the level of steady‐state ventilation: when VA is high, then plant gain is low which means for a given change in ventilation there will only be a small change in pACO_2_ (i.e., pACO_2_ fluctuations are dampened) and vice versa.

In the absence of upper airway obstruction, steady‐state VA is determined by the intersection between the chemosensitivity line and the isometabolic curve (termed eupneic alveolar ventilation; VA_eupnea_). The reciprocal slope of the tangent at this point thus reflects the (instantaneous) plant gain at VA_eupnea_. Note that the alveolar and arterial CO_2_ are approximately equal, in the following, we used the term paCO_2_ throughout for simplicity.

#### 2.1.3 Other parameters

The intersection between the chemosensitivity line and the *x*‐axis (VA = 0) is termed apnea threshold (i.e., the paCO_2_ at which ventilation stops), and the difference between the paCO_2_ at VA_eupnea_ and the paCO_2_ at the apnea threshold is known as CO_2_ reserve. Similarly, VA_reserve_ denotes the change in ventilation from VA_eupnea_ which will reduce the paCO_2_ to the level at which ventilation ceases (i.e., paCO_2_ at the apnea threshold). Note that the isometabolic curve and chemosensitivity line are the primary components that determine all these other parameters.

### 2.2 Data collection and meta‐analyses

For details, see Schmickl et al. (2020). In brief, we queried MEDLINE, EMBASE, and clinicaltrials.gov for any study which assessed the effect of oral acetazolamide in adult OSA/CSA patients versus a control condition (e.g., no acetazolamide or placebo) prior to November 03, 2019. Two reviewers independently assessed eligibility and abstracted data from included studies.

To assess the effect of acetazolamide on parameters of breathing control as a *relative* change from the control conditions, we used meta‐analyses based on ratio of means (ROM; results from sensitivity analyses based on *absolute* “weighted mean differences” were similar); Friedrich et al., 2008). Heterogeneity was quantified using *I* ^2^ which denotes the percentage of total variation across studies that is due to heterogeneity rather than chance (range: 0%–100%); (Higgins et al., 2003) For *I* ^2^ > 30%, we used random effects models and explored possible sources of heterogeneity (e.g., acetazolamide dose) via meta‐regression and/or qualitative assessments depending on the number of studies.

### 2.3 Model simulations

To identify potential predictors of response to acetazolamide, we created an *E*xcel‐based model of steady‐state *c*ontrol *o*f *b*reathing (“ECOB‐Model,” available for free download at: https://tinyurl.com/ECOB‐Model) using results from the meta‐analyses for CO_2_ production, paCO_2_ at VA_eupnea_ and the apnea threshold as inputs and estimating the percent change in loop gain (%ΔLG_0_) as the primary output:

$$\%\Delta {\text{LG}}_{0}=\left[\left(1+\%\Delta \text{CG}/100\right)\times \left(1+\%\Delta \text{PG}/100\right)‐\;1\right]\times 100,$$

where CG, controller gain and PG, plant gain).

Varying one input parameter (*i*) at a time across a physiological range (i.e., controller gain 0.5–3 L/min/mmHg, paCO_2_ at VA_eupnea_ 30–50 mmHg, CO_2_ production 155–255 ml/min), we then assessed the effect of different baseline values (*j*) on the percent change in loop gain (%ΔLG*_i_*_,_ *_j_*). To assess the relative effect of different baseline conditions on the change in loop gain, results of these simulations were indexed to the change in loop gain under the initial conditions (%ΔLG_0_) and plotted as a “relative reduction” (RR):

$${\text{RR}}_{i,j}=\%\Delta {\text{LG}}_{i,j}/\%\Delta {\text{LG}}_{0},$$

where %ΔLG_0_ was −21.8%, thus RR*_i_*_,_ *_j_* > 1 denotes a greater reduction in LG, whereas 0 < RR*_i_*_,_ *_j_* < 1 denotes a lesser reduction in LG (note: RR*_i_*_,_ *_j_* < 0 would reflect an increase in LG but was not observed).

## 3 RESULTS

We included data from 18 studies as shown in Table 1 (Apostolo et al., 2014; Caravita et al., 2015; DeBacker et al., 1995; Edwards et al., 2012; Fischer et al., 2004; Fontana et al., 2011; Ginter et al., 2020; Hackett et al., 1987; Javaheri, 2006; Pranathiageswaran et al., 2014; Rodway et al., 2011; Tojima et al., 1988; Ulrich et al., 2015; Verbraecken et al., 2005; Wellman, 2018; White et al., 1982) Acetazolamide dose ranged from 250 to 1000 mg/day and was administered for 1–30 days.

**TABLE 1:** Overview of included studies

	Population				Acetazolamide/Control						Outcomes										
	Type of sleep apnea	Mean age (years)	% Female	Mean BMI (kg/m^2^)	Dose (mg/day)	No. days	*N*_AZM_	*N*_Control_	Design	Control type	CO_2_ production	VRCO_2_	VRO_2_	Plant gain	Loop gain	Minute ventilation	Tidal volume	Respiratory rate	paCO_2_ VA_eupnea_	Apnea threshold	CO_2_ reserve
White et al. (1982)	CSA‐ID	58	0		750	2	9	9	OBS	Baseline									X^b^, ^c^		
Hackett et al. (1987)	CSA‐HA	31	0		750	1	4	4	RCT	Placebo			X^j^								
Tojima et al. (1988)	OSA	58	44.4	29.9	250	7.5	9	9	OBS	Baseline		X^a^	X^k^						X^b^, ^c^		
DeBacker et al. (1995)	CSA‐ID	48	7.1	31.5	250	30	14	14	OBS	Baseline		X^a^							X^b^, ^c^		
Fischer et al. (2004)	CSA‐HA		0		500	4	10	10	RCT	Placebo									X^b^, ^c^		
Verbraecken et al. (2005)	CSA‐ID	57		38.3	250	1	39	39	OBS	Baseline									X^b^, ^c^		
Javaheri, (2006)	CSA‐CHF	66	0	26	281^i^	6	12	12	RCT	Placebo	X	X^a^				X^b^	X^b^	X^b^	X^b^, ^c^		
Rodway et al. (2011)	CSA‐HA	38			125	1	4	4	RCT	No AZM						X	X	X			
Fontana et al. (2011)	CSA‐CHF	62	8.3	29	500	4	12	12	OBS	Baseline		X^a^	X^k^						X^b^, ^c^		
Edwards et al. (2012)	OSA	50		34.2	1000	6.5	12	12	RCT	Baseline		X^g^		X	X	X	X	X	X^d^		
Nussbaumer‐Ochsner et al. (2012)	OSA (+HA)	64	6.7	31.7	500	3	45	45	RCT	Placebo									X^e^	X	X
Latshang et al. (2012)	CSA‐HA	63	5.9	33	750	3	51	51	RCT	Placebo									X^e^		
Apostolo et al. (2014)	OSA (+CHF)	69	0	24.5	1000	2	18	18	OBS	Baseline	X					X^b^	X^b^	X^b^	X^b^, ^c^		
Pranathiageswaran et al. (2014)	OSA	56	75	28	1000	3	4^f^	4	RCT	Placebo		X^h^		X		X				X	X
Caravita et al. (2015)	CSA‐HA	36	48.8	21.9	500	4	20	17	RCT	Placebo						X^b^			X^b^, ^d^		
Ginter et al. (2020)	CSA‐Other	55		25.5	1000	3	14	14	RCT	Placebo		X^h^		X		X	X	X		X	X
Ulrich et al., (2015)	CSA‐Other	66	65.2	26.6	500	7	23	23	RCT	Placebo									X^b^, ^c^		
NCT01377987 Wellman (2018)	OSA (+CHF)	60	10.3		300	7	22	22	RCT	Placebo					X						

Abbreviations: AZM, acetazolamide; BMI, body mass index; CSA, central sleep apnea; CHF, congestive heart failure; HA, high altitude; ID, idiopathic; OBS, observational study; RCT, randomized controlled trial; VRCO_2_, ventilatory response to CO_2_; VRO_2_, ventilatory response to O_2_; VA_eupnea_ denotes alveolar ventilation when there is no airway obstruction (ventilation = demand).

^a^ Measured using Read's rebreathing technique (awake subjects).

^b^ Measured while subjects were awake.

^c^ Arterial blood gas.

^d^ End‐tidal CO_2_.

^e^ Transcutaneous CO_2_.

^f^ For CO_2_ reserve, N_AZM_ = 2.

^g^ During stable NREM sleep, subjects were placed on therapeutic levels of CPAP, with intermittent 3‐min drops to subtherapeutic pressures. Loop gain (LG) was calculated as the mean of the ventilatory response divided by the ventilatory disturbance at the end of each 3‐min pressure drop. Plant gain (PG) was calculated as the reciprocal of the metabolic hyperbola during NREM sleep. Finally, controller gain (≈ VRCO_2_) = LG/PG (Edwards et al., 2012).

^h^ During stable NREM sleep, subjects were placed on therapeutic levels of CPAP. Intermittently, pressure support was added for 3‐min periods resulting in a sustained decrease of P_ET_CO_2_. Pressure support (PS) was initiated at 8 cm H_2_O and gradually increased 2 cm H_2_O (across these 3‐min periods) until a trial of hyperventilation resulted in a central apnea. The five breaths before PS initiation were designated as the control breaths, whereas the five last breaths prior to central apnea were designated as mechanical ventilation (MV) breaths. Finally, controller gain (≈VRCO_2_) = (MinuteVentilation_Control_–MinuteVentilation_Post‐MV_)/(P_ET_CO_2, Control_−P_ET_CO_2,MV_). Note, for rare subjects with spontaneous central apneas, a similar protocol was used by applying gradually increased concentrations of CO_2_ (instead of pressure support ventilation) until apneas resolved (Ginter et al., 2020; Pranathiageswaran et al., 2014).

^i^ Subjects received 3.5 to 4 mg/kg/day. Assuming an average weight of 75 kg, we estimated the mean daily dose as 75 kg x 3.75 mg/kg/day = 281 mg/day.

^j^ VRO_2_ was calculated as the linear regression slope between ventilation and oxygen saturation during a progressive poikilocapnic hypoxia trial (from resting SaO_2_ values >95% to a minimum of 50%, according to individual tolerance) which was indexed to body surface area.

^k^ VRO_2_ was calculated as the linear regression slope between ventilation and oxygen saturation during a progressive isocpanic hypoxia trial (from resting SaO_2_ values to 70%–80%, according to individual tolerance).

### 3.1 Meta‐analyses

**FIGURE 2:**
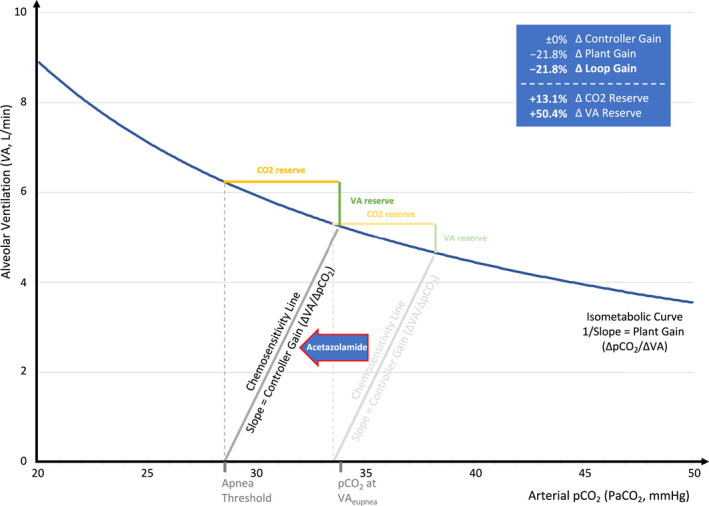
Effects of acetazolamide on control of breathing based on meta‐analyses. Assuming acetazolamide causes no change in CO_2_ production (i.e., no change in isometabolic curve) and chemosensitivity slope, but a left shift of apnea threshold by 15% (i.e., −5 mmHg, based on the baseline conditions described in Figure 1). Note that eupneic ventilation occurs at a steeper portion of the isometabolic curve (i.e., lower plant gain) and that the reduction in paCO_2_ at eupneic ventilation (−4.4 mmHg; −11%) is less than the reduction of the apnea threshold, thus increasing the CO_2_ and ventilatory (VA) reserves

Table 2 and Figure 2 summarize acetazolamide's effects on the ventilatory control system based on several meta‐analyses, which are described below.

**TABLE 2:** Effects of acetazolamide on ventilatory control parameters based on meta‐analyses

			*N*_Studies_		MD (95% CI)	Control		
	ROM (95% CI)	*I* ^2^	(*N*_OSA|_ *N*_CSA_)	P_Δ=0_	(ROM × Mean_Wt_–Mean_Wt_)	Mean_wt_	SD_wt_	*N*_Subj_
Isometabolic curve								
CO_2_ production (ml/min)	1.09 (0.97–1.23)	0%	2 (1|1)	0.13	+18.5 (−6.2 to 47.4)	206	(36.9)	26
Chemosensitivity line								
VRCO_2_ (L/min/mmHg)^a^	1.06 (0.87–1.28)	37%	7 (3|4)	0.59	+0.1 (−0.3 to 0.6)	2.14	(0.97)	70
Read's technique, awake	1.31 (1.05–1.63)	0%	4 (1|3)	0.02*	+0.6 (0.1 to 1.2)	1.92	(1.0)	40
Other techniques, asleep	0.84 (0.70–1.02)	0%	3 (2|1)	0.08	−0.39 (−0.73 to 0.05)	2.44	(0.84)	30
VRO_2_ (L/min/%SaO_2_)^b^	1.0 (0.69–1.43)	35%	3 (1|2)	0.98	0 (−0.2 to 0.3)	0.68	(0.42)	23
Apnea threshold (mmHg)	0.85 (0.79–0.91)	0%	3 (2|1)	<0.001*	−5.1 (−7.1 to −3.0)	33.5	(5.4)	36
paCO_2_ at VA_eupnea_ (mmHg)^c^	0.89 (0.86–0.92)	75%	13 (4|9)	<0.001*	−4.2 (−5.5 to −2.9)	38.2	(3.7)	265
<500 mg/day	0.93 (0.89–0.98)	45%	4 (1|3)	0.007*	−2.7 (−4.5 to −0.7)	39.0	(2.6)	65
≥500 mg/day	0.87 (0.84–0.92)	77%	9 (3|6)	<0.001*	−4.8 (−6.3 to −3.2)	37.8	(4.1)	200
Minute ventilation (L/min)^d^	1.13 (1.05–1.22)	0%	7 (3|4)	0.001*	+1.2 (0.5 to 2)	9.1	(2.5)	83
Tidal volume (ml)^d^	1.24 (1.19–1.29)	3%	5 (2|3)	<0.001*	+132 (105 to 161)	562.1	(57.5)	58
Respiratory rate (min^−1^)^d^	0.99 (0.96–1.02)	11%	5 (2|3)	0.49	−0.2 (−0.7 to 0.4)	16.0	(1.2)	58
Other parameters								
Plant gain (mmHg/L/min)	0.68 (0.57–0.82)	0%	3 (2|1)	<0.001*	−1.7 (−2.3 to −1)	5.42	(1.78)	30
Loop gain^e^ (dimensionless)	0.74 (0.55–1.0)	42%	2 (2|0)	0.049*	−0.1 (−0.2 to 0)	0.52	(0.22)	40
CO_2_ Reserve (mmHg)^f^	1.53 (1.1–2.2)	71%	3 (2|1)	0.02*	+2.1 (0.3 to 4.6)	4.0	(1.6)	36

Abbreviations: ROM, ratio of means; MD, mean difference; VRCO_2_, ventilatory response to CO_2_; VRO_2_, ventilatory response to O_2_; VA_eupnea_ denotes alveolar ventilation when there is no airway obstruction (ventilation = demand); Mean_wt_/SD_wt_, weighted mean and standard deviation in the control groups (using weights from the meta‐analysis).

^a^ Heterogeneity was explained by different techniques: VRCO_2_ was significantly increased in the studies (DeBacker et al., 1995; Javaheri, 2006; Tojima et al., 1988) using Read's Rebreathing technique (considered invalid in the setting of acetazolamide) but did not significantly change in studies (Edwards et al., 2012; Fontana et al., 2011; Ginter et al., 2020; Pranathiageswaran et al., 2014) using other techniques (see text for more details). Dose was considered as an effective modifier but was collinear with the technique (i.e., Read technique <500 mg, other techniques >500 mg/day).

^b^ One study (Hackett et al., 1987) assessed VRO_2_ under poikilocapnic conditions and indexed the response to the body surface area (L/min/m^2^/%SaO_2_), whereas the other two studies (Fontana et al., 2011; Tojima et al., 1988) assessed VRO_2_ under isocapnic conditions without accounting for body surface area (L/min/%SaO_2_). When excluding the former study in a sensitivity analysis, then heterogeneity resolved and there was a reduction of VRO_2_ by 24%, but results were nonsignificant with a wide confidence interval (ROM = 0.76, 95% CI: 0.48–1.20, *I* ^2^ = 0%, *p* = 0.24).

^c^ No clear cause of heterogeneity was identified: results were similar in studies using ABGs versus other tests to estimate paCO_2_ as well as in studies that measured paCO_2_ during wakefulness versus during sleep. Meta‐regression suggested a dose–response relationship and the paCO_2_ reduction was almost twice as large in studies administering ≥500 mg/day versus <500 mg/day (reduction by 13% vs. 7%) but these differences did not reach statistical significance (*p* > 0.18).

^d^ In sensitivity analyses, results were similar in studies that performed measurements during wakefulness versus sleep.

^e^ When using standardized mean differences, results were similar but heterogeneity resolved (SMD = −0.68, 95 CI −1.13 to −0.23 [corresponding to a loop gain reduction by 29%, 95% CI −48 to −10]; *I* ^2^ = 0%, *p* = 0.003) suggesting that heterogeneity was due to different measurement scales (mean loop gain across various frequencies ranging from 0.5 to 1.5/min^2^ vs. loop gain at a frequency of 1 per minute (Wellman, 2018); results were also similar when using the static instead of dynamic loop gain reported by Edwards et al. (2012).

^f^ Heterogeneity was primarily due to one small study (Pranathiageswaran et al., 2014) (*N*_control_ = 4, *N*_AZM_ = 2) in which the acetazolamide‐induced increase in CO_2_ reserve was about two times larger as in other studies with a very small reported standard deviation. In sensitivity analyses, results were similar to minimal heterogeneity when excluding this study (1.25 [95% CI 1.04–1.5], *I* ^2^ = 0%, *p* = 0.01) or when assuming that this study erroneously reported standard errors instead of standard deviations (1.30 [95% CI 1.08–1.6], *I* ^2^ = 17%, *p* = 0.005).

* denotes *p* < 0.05.

#### 3.1.1 Isometabolic curve

Acetazolamide increased CO_2_ production by 9% (95% CI: −3 to +23; *p* = 0.13, *N* = 2 [Apostolo et al., 2014; Javaheri, 2006]), perhaps in part due to increased respiratory work, but the estimate is imprecise (Lederer et al., 2019). Based on the confidence interval, these results are compatible with no change, or an increase of the slope, of the isometabolic curve in response to acetazolamide (i.e., the same or lower plant gain for any given pACO_2_).

#### 3.1.2 Chemosensitivity line

Based on seven studies (DeBacker et al., 1995; Edwards et al., 2012; Fontana et al., 2011; Ginter et al., 2020; Javaheri, 2006; Pranathiageswaran et al., 2014; Tojima et al., 1988) (all performed at low altitude, see Table 1) acetazolamide did not significantly affect VRCO_2_ (+6%, *p* = 0.59), but there was moderate heterogeneity (*I* ^2^ = 37%), which was likely explained by methodological differences: VRCO_2_ increased by 31% (*I* ^2^ = 0%, *p* = 0.02) in the four studies (DeBacker et al., 1995; Fontana et al., 2011; Javaheri, 2006; Tojima et al., 1988) using Read's rebreathing technique that assesses response to hyperoxic hypercapnia in wakefulness but is considered invalid in the setting of acetazolamide (the greater initial step change in end‐tidal pCO_2_ in the acetazolamide condition artificially increases the slope measured by Read's rebreathing technique) (Teppema & Dahan, 1999); in the three studies, (Edwards et al., 2012; Ginter et al., 2020; Pranathiageswaran et al., 2014) assessing VRCO_2_ via other techniques during sleep under poikiloxic conditions (for details about these techniques, see footnotes of Table 1), there was no statistically significant change (−16%; *I* ^2^ = 0%; *p* = 0.08), but the confidence interval was compatible with a decrease in VRCO_2_ by as much as 30%.

Similarly, acetazolamide did not significantly affect VRO_2_, although there was some unexplained heterogeneity and the confidence interval was wide (−0.5%; 95% CI: −31% to +43%; *I* ^2^ = 35%; *p* = 0.98, *N* = 3 [Fontana et al., 2011; Hackett et al., 1987; Tojima et al., 1988]). These data suggest that under normoxic conditions acetazolamide does either not change or perhaps reduce the slope of the chemosensitivity line (i.e., no change, or reduction, of the controller gain).

Further, acetazolamide reduced the apnea threshold (i.e., the *x*‐intercept of the chemosensitivity line) by 15% (*p* < 0.001, *N* = 3 [Ginter et al., 2020; Pranathiageswaran et al., 2014; Nussbaumer‐Ochsner et al., 2012]) and reduced paCO_2_ at VA_eupnea_ by a similar magnitude (−11%; *p* < 0.001, *N* = 13 [Apostolo et al., 2014; Caravita et al., 2015; DeBacker et al., 1995; Edwards et al., 2012; Fischer et al., 2004; Fontana et al., 2011; Javaheri, 2006; Latshang et al., 2012; Nussbaumer‐Ochsner et al., 2012; Tojima et al., 1988; Ulrich et al., 2015; Verbraecken et al., 2005; White et al., 1982]). In combination, these data suggest a left shift of the chemosensitivity line and indirectly support the notion that acetazolamide does not increase the slope of the chemosensitivity line.

VA_eupnea_ was not directly measured in any of the included studies. However, acetazolamide increased eupneic minute ventilation by 13% (*p* = 0.001, *N* = 7 [Apostolo et al., 2014; Caravita et al., 2015; Edwards et al., 2012; Ginter et al., 2020; Javaheri, 2006; Pranathiageswaran et al., 2014; Rodway et al., 2011]). Additional meta‐analyses suggested that this increase is due to an increase in tidal volume (+23%, *p* < 0.001, *N* = 5 (Apostolo et al., 2014; Edwards et al., 2012; Ginter et al., 2020; Javaheri, 2006; Rodway et al., 2011)) without change in respiratory rate (−1%; *p* = 0.49, *N* = 5 (Apostolo et al., 2014; Edwards et al., 2012; Ginter et al., 2020; Javaheri, 2006; Rodway et al., 2011). Together, these data suggest that dead space ventilation remains unchanged while alveolar ventilation at eupnea (i.e., VA_eupnea_) increases by 5%–29% (conservative estimate based on the outer limits of the 95% CIs for minute ventilation and tidal volume).

#### 3.1.3 Loop/plant gain

Acetazolamide decreased plant gain by 32% (*p* < 0.001, *N* = 3 [Edwards et al., 2012; Ginter et al., 2020; Pranathiageswaran et al., 2014]) with a similar decrease in overall loop gain by 26% (*p* = 0.049, *N* = 2 [Edwards et al., 2012; Wellman, 2018]). Of note, the similar magnitude of change in plant and overall loop gain provides further indirect evidence that the slope of the chemosensitivity line (i.e., controller gain) does not change.

#### 3.1.4 CO_2_ reserve

Acetazolamide increased the CO_2_ reserve by 53% (*p* = 0.02, *N* = 3 [Ginter et al., 2020; Nussbaumer‐Ochsner et al., 2012; Pranathiageswaran et al., 2014]), but heterogeneity was high (*I* ^2^ = 71%) primarily due to one small study (Pranathiageswaran et al., 2014) (N_Subjects, Control_ = 4, N_Subjects, Acetazolamide_ = 2) in which the acetazolamide‐induced increase in CO_2_ reserve was about two times larger as in other studies with a very small reported standard deviation. In sensitivity analyses, the increase in CO_2_ reserve was more modest but remained statistically significant when excluding this study (Pranathiageswaran et al., 2014) (+25%, *I* ^2^ = 0%, *p* = 0.01) or when assuming that this study erroneously reported standard errors instead of standard deviations (1.30 [95% CI 1.08–1.6], *I* ^2^ = 17%, *p* = 0.005).

### 3.2 Model simulations

As shown in Figure 1, we first modeled the isometabolic curve and the chemosensitivity line using pooled data from control conditions (Table 2) as inputs for the ECOB‐Model (i.e., assuming CO_2_ production = 206 ml/min, paCO_2_ at VA_eupnea_ = 38.2 mmHg, and paCO_2_ at the apnea threshold = 33.5 mmHg). Next, based on the results from meta‐analyses, we modeled a left shift of the apnea threshold by 15% (i.e., −5 mmHg) without change in the slope of the chemosensitivity line (i.e., a left shift of the chemosensitivity line; Figure 2). The predicted changes in loop gain and other model output parameters were well within the range of the 95% CIs from meta‐analyses providing face validity for this model simulation (see Table 3, Model 1). Alternative models assuming additionally an increase in CO_2_ production by 9% and/or a reduction of controller gain by 11% showed similar results although the predicted reductions in loop gain were more pronounced (Table 3, Models 2–4).

**TABLE 3:** Model predictions compared with estimates from meta‐analyses

	95% CI from meta‐analyses	Primary model Model 1	Sensitivity analyses		
			Model 2 Apnea threshold −15% and CO_2_ production +9%	Model 3 Apnea threshold −15% and controller gain −11%	Model 4 Apnea threshold −15% and CO_2_ production +9% and controller Gain −11%
		Apnea threshold −15%			
VA_eupnea_ (L/min)	+5% to +29%^a^	+13.1%	+21.8%	+11.2%	+19.7%
paCO_2_ at VA_eupnea_ (mmHg)	−14% to −8%	−11.5%	−10.5%	−10.1%	−8.9%
CO_2_ reserve (mmHg)	+7% to +117%	+13.1%	+21.8%	+25.0%	+34.5%
VA reserve (L/min)	na	+50.4%	+74.4%	+63.5%	+89.3%
Plant gain (mmHg/L/min)	−43% to −18%	−21.8%	−26.5%	−19.2%	−23.9%
Loop gain	−45% to 0%	−21.8%	−26.5%	−28%	−32.3%

^a^ Based on the outer limits of the 95 CIs of pooled estimates for minute ventilation and tidal volume.

**FIGURE 3:**
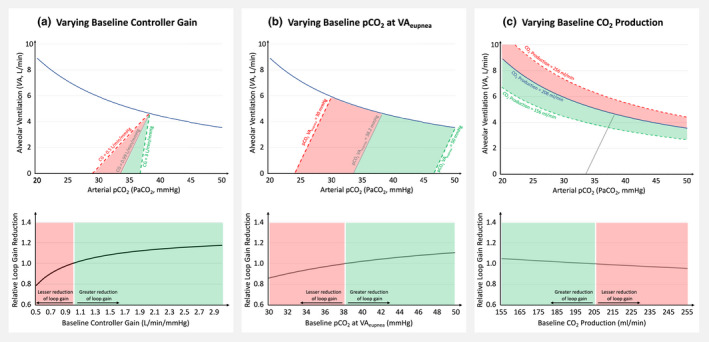
Model simulations: The impact of varying baseline Conditions (a–c) on the relative reduction of loop gain induced by acetazolamide. Top panels demonstrate the range of simulation, bottom panels show the relative reduction in loop gain compared with the initial condition (see Methods for details)

**FIGURE 4:**
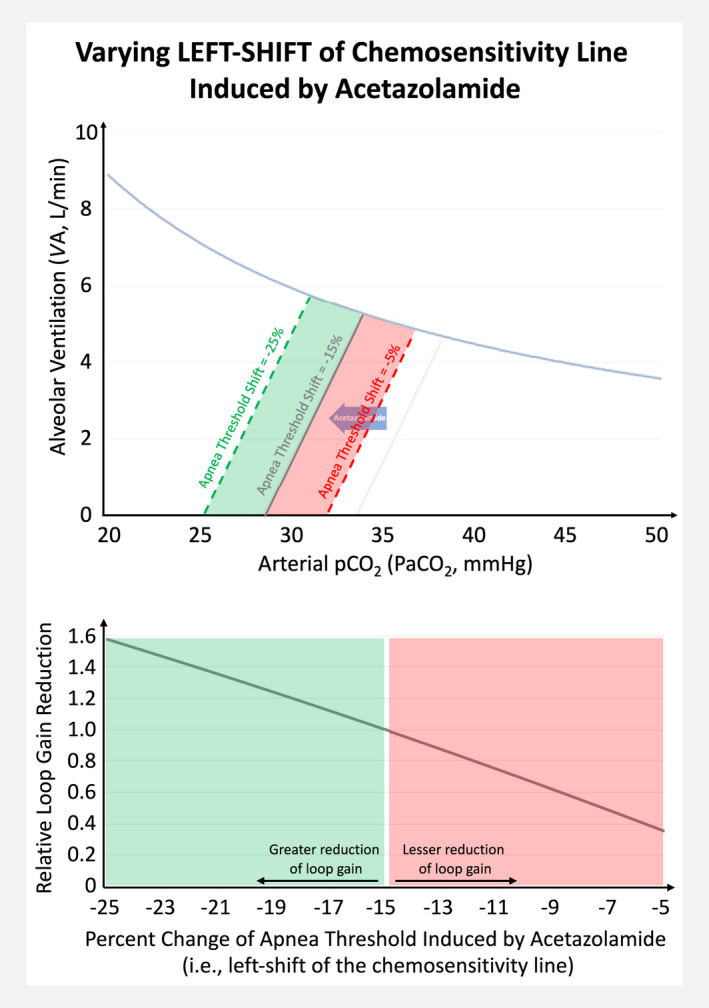
Model simulation: The impact of varying left shifts of the chemosensitivity line on the relative reduction of loop gain induced by acetazolamide

Based on Model 1, we then assessed how the effect of acetazolamide on loop gain is modified by different baseline conditions (Figure 3): the loop gain reduction induced by acetazolamide was more pronounced with higher controller gain (up to ~20% greater reduction), higher paCO_2_ at VA_eupnea_ (up to ~10% greater reduction), and with lower CO_2_ production at baseline (up to ~5% greater reduction). Importantly, the amount by which acetazolamide shifts the chemosensitivity line to the left is a major determinant of the resulting change in loop gain (Figure 4).

## 4 DISCUSSION

Strengths of our study include the simultaneous examination of acetazolamide's effects on the different components of ventilatory control and the combination of meta‐analyses and physiological model simulations to gain several, important mechanistic insights.

First, to our knowledge, this is the first comprehensive, mechanistic meta‐analysis of acetazolamide's effect on ventilatory control based on several small studies, thus providing a unified framework to explain how this drug stabilizes breathing (i.e., lowers loop gain) in patients with sleep apnea: our results suggest that acetazolamide primarily shifts the chemosensitivity line to the left. Thus, eupneic ventilation occurs at a steeper part of the isometabolic curve which results in a lower plant gain. Loop gain is proportional to controller and plant gain, thus under normoxic conditions (in which there appears to be no substantial change in controller gain), the relative reduction in plant gain is equal to the reduction in loop gain. Of note, we have previously shown that acetazolamide increases pO_2_ on average by ~10 mmHg, (Schmickl et al., 2020) thus under hypoxic conditions, such as high altitude, one would additionally expect a reduction in controller gain (i.e., mitigation of the hypoxia‐related increase in controller gain), and thus a more pronounced reduction in overall loop gain. Studies comparing acetazolamide's effect on controller/loop gain at different altitudes are lacking, but these theoretical considerations are supported by empirical data showing that acetazolamide improves sleep apnea more at high versus low altitude (Schmickl et al., 2020). The control of breathing model that we used provides another, perhaps more intuitive way to understand the effects of acetazolamide: because the isometabolic curve is hyperbolic, the left‐shift of the chemosensitivity line lowers the apnea threshold more than the paCO_2_ at eupneic ventilation (e.g., −5 mmHg vs. −4.4 mmHg, Figure 2) thus increasing the CO_2_ and ventilatory reserves. In other words, a subject taking acetazolamide needs to increase ventilation and blow off more CO_2_ to reach the apnea threshold than without acetazolamide, thus reducing the risk of developing central hypopneas and apneas which tend to also lead to upper airway collapse (i.e., can directly contribute to obstructive events; Badr et al., 1995).

Second, based on model simulations, we identified baseline controller gain and—to a lesser extent—baseline paCO_2_ at eupneic ventilation as physiological predictors of the loop gain reduction achieved by acetazolamide. This may explain the pronounced improvement of sleep apnea in patients with heart failure (who tend to have a high controller gain) which appears to be less than the improvement of high altitude sleep apnea, but greater than other types of sleep apnea (see Figure 3 in Schmickl et al., [2020]). Further, given the linear relationship between loop gain reduction and reduction of the apnea‐hypopnea index in small studies, (Terrill et al., 2015) these predictors may facilitate enrollment of likely responders into future trials. For example, paCO_2_ at eupneic ventilation and controller gain can be estimated via end‐tidal CO_2_ measurements during a baseline study; (Edwards et al., 2012) using our online ECOB‐calculator the expected reduction in loop gain can then be estimated (https://tinyurl.com/ECOB‐Model).

Third, our simulations also demonstrated that the amount of the left‐shift of the chemosensitivity line is a critical determinant of the achieved loop gain reduction. Our meta‐analyses suggested that the degree of left shift is dose dependent (there were insufficient studies to assess for dose effects on the apnea threshold, but the paCO_2_ at VA_eupnea_ decreased by 7% and 13% in studies administering <500 mg/day and ≥500 mg/day, respectively; Table 2). This left shift likely reflects the increased eupneic ventilation caused by the acetazolamide‐induced metabolic acidosis acting on peripheral and central chemoreceptors (Swenson, 1998). The metabolic acidosis from acetazolamide that reaches its maximum within 24 h of administration is primarily due to an alkaline diuresis via renal carbonic anhydrase inhibition but local tissue acidification can contribute too (Swenson, 1998). Importantly, most of these effects are maximal at 250–500 mg, (Swenson, 1998), which likely explains the dose‐response relationship between acetazolamide and the improvement of sleep apnea up to ~500 mg/day (after which the effects seem to plateau) in interventional studies (Schmickl et al., 2020).

A major limitation of our work is that the control of breathing model which we used assumes steady‐state conditions (vs. the dynamic conditions typically observed during OSA/CSA) and that the concept of loop gain describes behaviors of linear systems, whereas nonlinearities exist in the respiratory control system (Dempsey, 2019). However, experimental studies in humans and animals have repeatedly demonstrated that alterations of overall loop gain or its components, even when measured under steady‐state conditions, result in predictable changes in ventilatory stability and/or sleep apnea severity (Dempsey, 2019). Furthermore, in our analyses, we implicitly assumed that acetazolamide has no effect on the third component of loop gain, namely mixing gain which includes complex time constants and is rarely studied in the setting of acetazolamide. A major contributor to mixing gain is the time that it takes for the alveolar pACO_2_ to be transmitted to the chemoreceptors in the carotid body which is primarily a function of cardiac output; the normal “circulatory delay” is about 7 s (Younes, 2014), but in the heart failure patients, this time may increase substantially, thus increasing mixing gain and overall loop gain (Stanchina et al., 2007). Given its mild diuretic effects acetazolamide may improve cardiac output and thus yield a greater reduction in overall loop gain in patients with heart failure, but one would not expect much effect of acetazolamide on cardiac output in patients without heart failure. Another component of mixing gain is the response speed of the different chemoreceptors which appears to be unaffected by acetazolamide based on experimental data (Teppema & Dahan, 1999). There were a few other noteworthy limitations: First, meta‐analyses for the different ventilatory control components included different studies which had some methodological variability. But reassuringly most analyses revealed only a small amount of statistically detectable heterogeneity (i.e., *I* ^2^ < 30–50%), and results were overall consistent with patient‐level data reported in many individual studies as well as with what is expected based on physiological models (Table 3). Second, by excluding studies in subjects without sleep apnea, we increased internal validity but reduced the sample size of studies included in meta‐analyses and thus the precision of our results. However, confidence intervals for most results were sufficiently narrow to draw firm conclusions. Small sample sizes in most meta‐analyses also limited our ability to assess for effect modification by study characteristics such as acetazolamide dose or duration of administration. A notable exception is the meta‐analysis of paCO_2_ at VA_eupnea_, which did suggest that doses ≥500 mg result in a greater left shift of the chemosensitivity line than doses <500 mg/day, which may explain the dose–response relationship between acetazolamide and the improvement of sleep apnea severity (up to ~500 mg/day) (Schmickl et al., 2020). Regarding the duration of administration, acetazolamide is expected to take full effect within 24 h (Swenson, 1998), and results were similar in sensitivity analyses excluding the three studies (Hackett et al., 1987; Rodway et al., 2011; Verbraecken et al., 2005) in which acetazolamide was administered for <2 days (data not shown). Similarly, in exploratory analyses, there was no apparent effect modification by sleep apnea type or percentage of women (data not shown) but results from one study (Caravita et al., 2015) did suggest a greater left shift in men than women. Thus, the low percentage of women in most prior studies limits the generalizability of our findings and we advocate for greater inclusion of women in future research. Finally, our data and analyses were performed with the objective of predicting change in loop gain with acetazolamide, not the change in OSA or CSA severity, which is affected by other endotypic traits as well (Owens et al., 2015; Schmickl et al., 2018).

Prospective studies are needed to assess better the relationships between acetazolamide dose, the induced metabolic acidosis, and the achieved left shift of the chemosensitivity line. More research is also needed to assess how accurately the presented online calculator predicts changes in loop gain in individual patients and to validate baseline controller gain and paCO_2_ at baseline as predictors of the loop gain reduction.

## 5 CONCLUSION

Using a meta‐analysis approach, we were able to demonstrate the impact of acetazolamide on the control of ventilation and more precisely estimate its impact: acetazolamide primarily causes a left shift of the chemosensitivity line but, in general, does not substantially affect CO_2_ production or controller gain. An elevated baseline controller gain and paCO_2_ at eupneic ventilation may predict greater reductions in loop gain from acetazolamide. Ultimately, the combination of physiological and other patient characteristics may allow highly accurate identification of patients responding to loop gain lowering interventions facilitating a personalized medicine approach.

## CONFLICT OF INTERESTS

The following is a list of general funding sources: Dr. Schmickl received salary support from NHLBI (T32 grant HL134632 “Training the Next Generation in Respiratory Science”) and the ATS Foundation during the conduct of the study. Dr. Nokes is supported by the NIH (T32 grant HL134632), Sleep Research Society Career Development Award, as well as the American Thoracic Society ASPIRE Fellowship. Dr. Landry and Dr. Orr have nothing to disclose. Dr. Edwards reports grants from Heart Foundation of Australia during the conduct of the study, grants from National Health and Medical Research Council, grants from Apnimed, and personal fees from Signifier Medical, outside the submitted work. Dr. Owens is funded by the NHLBI (HL142114), and reports personal fees from Novartis and Nitto Denko Asia, outside the submitted work. Dr. Malhotra is funded by NHLBI. He received income from Livanova, Corvus, and Equillium for medical education. ResMed provided a philanthropic donation to UC San Diego.

## AUTHOR CONTRIBUTIONS

Study design: CS, AM, RO; Data acquisition: CS, SL, AM; Data Analysis: CS; Data interpretation: All; Draft: CS; Revisions: All. All authors approved the final version of the manuscript; agree to be accountable for all aspects of the work in ensuring that questions related to the accuracy or integrity of any part of the work are appropriately investigated and resolved; and all persons designated as authors qualify for authorship, and all those who qualify for authorship are listed.

## Data Availability

Any available data are included as a citation in the references section.
